# Properties and Functions of Feline Immunodeficiency Virus Gag Domains in Virion Assembly and Budding

**DOI:** 10.3390/v10050261

**Published:** 2018-05-16

**Authors:** Silvia A. González, José L. Affranchino

**Affiliations:** Laboratorio de Virología, CONICET-Universidad de Belgrano, Villanueva 1324, Buenos Aires C1426BMJ, Argentina; silvia.gonzalez@comunidad.ub.edu.ar

**Keywords:** feline immunodeficiency virus, Gag polyprotein, retrovirus assembly, retrovirus budding

## Abstract

Feline immunodeficiency virus (FIV) is an important cat pathogen worldwide whose biological and pathophysiological properties resemble those of human immunodeficiency virus type 1 (HIV-1). Therefore, the study of FIV not only benefits its natural host but is also useful for the development of antiviral strategies directed against HIV-1 infections in humans. FIV assembly results from the multimerization of a single but complex viral polypeptide, the Gag precursor. In this review, we will first give an overview of the current knowledge of the proteins encoded by the FIV *pol*, *env*, *rev*, *vif,* and *orf-A* genes, and then we will describe and discuss in detail the critical roles that each of the FIV Gag domains plays in virion morphogenesis. Since retroviral assembly is an attractive target for therapeutic interventions, gaining a better understanding of this process is highly desirable.

## 1. Introduction

Feline immunodeficiency virus (FIV) is a lentivirus that induces an AIDS-like syndrome in domestic cats [1]. Although FIV also infects wild felids, they appear not to develop feline AIDS [2]. However, there is evidence suggesting that FIV is a potentially harmful agent in free-ranging lions [3]. FIV is mainly transmitted by bite wounds during fight or coitus [4]. In addition, mother-to-offspring FIV exposure can occur both in utero and postnatally [5,6]. Of note, FIV has a broader tropism than that of HIV-1, since it not only infects CD4^+^ T lymphocytes, monocytes, and macrophages but also exhibits tropism for CD8^+^ T and B lymphocytes [7,8,9,10]. FIV shows structural, genomic, biochemical, and pathophysiological similarities with human immunodeficiency virus type 1 (HIV-1) [5,11]. Indeed, infected domestic cats develop immune dysfunction, including a marked decline in CD4^+^ T lymphocytes, thymic depletion, and lymphoid hyperplasia, as well as susceptibility to opportunistic infections and rare cancers [5,12]. Therefore, FIV is not only an important cat pathogen but also a useful model of HIV-1 infections in humans [5,11,12,13]. In this regard, FIV features have allowed to model in domestic cats certain aspects of HIV-1 infections: (a) transmission of cell-associated, as well as cell-free, viruses; (b) mucosal infectivity; (c) host immune responses; (d) acquired immunodeficiency; (e) thymic dysfunction; (f) neuropathogenesis; (g) host-virus interactions; and (h) efficacy of antiviral therapy and experimental vaccines [12]. Indeed, a wealth of data characterizing FIV infection of cats has provided relevant biological information translatable to HIV-1 [5,12,13,14]. Furthermore, FIV-infected cats were the first animal model used to test HIV-1 reverse transcriptase inhibitors [15,16] and the efficacy of acyclic nucleoside phosphonate analogues, which led to the development of tenofovir, a common drug used for the treatment of HIV-1 infections [17,18]. Moreover, FIV is also sensitive to HIV-1 integrase inhibitors [19].

## 2. FIV Genome

**Figure 1:**
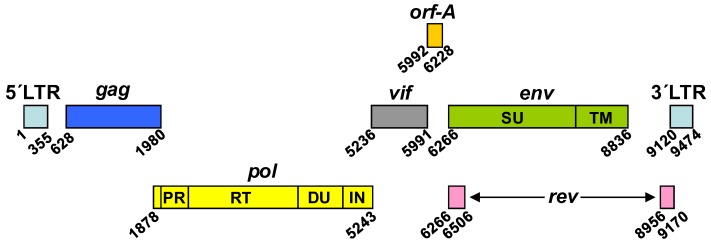
Organization of the FIV proviral DNA (Petaluma FIV-14 molecular clone; GenBank: M25381.1). The 5′ and 3′ long terminal repeats (LTR) are shown together with the open reading frames encoding Gag, Pol, Env, and the regulatory proteins Rev, Vif, and Orf-A. Numbers indicate the nucleotide positions in the FIV genome. PR, protease; RT, reverse transcriptase; DU, dUTPase; IN, integrase; SU, Env surface subunit; TM, Env transmembrane subunit.

FIV has a genome of around 9400 nucleotides, which is flanked by the Long Terminal Repeats (LTR) and contains the characteristic retroviral genes *gag*, *pol,* and *env* (Figure 1) [20]. In addition, the FIV genome encodes the regulatory proteins Rev, Vif, and OrfA (Figure 1) [20]. However, FIV lacks the accessory HIV-1 genes *vpr*, *vpu*, and *nef,* as well as *tat,* whose product regulates viral genome transcription [20].

## 3. FIV Proteins and Their Role in the Viral Life Cycle

### 3.1. Gag Polyprotein

FIV assembly, like that of all lentiviruses, occurs at the plasma membrane of the infected cells as the result of the multimerization of the Gag polyprotein into virions, which are then released into the extracellular medium. The FIV Gag polyprotein contains all the necessary information for virion assembly and budding [21]. Indeed, the expression in cell cultures of the FIV Gag precursor, in the absence of other viral proteins, results in the formation of particles morphologically similar to immature virions [21]. Moreover, recombinant FIV Gag expressed in *Escherichia coli* self-assembles in vitro into spherical particles, albeit of a smaller size (33 nm) than authentic virions [22]. Likewise, the Gag polyproteins of the primate lentiviruses HIV-1 and simian immunodeficiency virus (SIV) are capable of assembling into virus-like particles both in vivo and in vitro [23,24,25,26,27].

**Figure 2:**
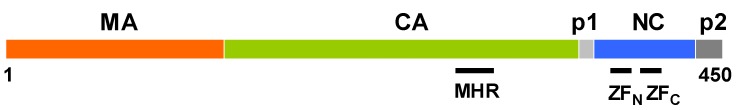
Domains of the FIV Gag polyprotein. The matrix (MA), capsid (CA), nuclecapsid (NC), spacer peptide p1, and C-terminal peptide p2 are shown. The location of the major homology region (MHR) in the CA, as well as that of the proximal (ZF_N_) and distal (ZF_C_) zinc finger motifs in the NC, are indicated. Numbers correspond to the first and last Gag residues.

Concomitantly with virion budding, the FIV Gag polyprotein is processed by the *pol*-encoded viral protease into the structural proteins of the mature particles: matrix (MA, 135 amino acids), capsid (CA, 222 residues), spacer peptide p1 (9 amino acids), nucleocapsid (NC, 66 residues), and the C-terminal p2 peptide (18 amino acids) [28] (Figure 2). A detailed description of the role of each of the FIV Gag domains will be discussed later in Section 4.

### 3.2. Pol

The FIV proteins encoded by the *pol* gene derive from a large Gag-Pol polyprotein, which is generated as a consequence of a −1 frameshift during translation of the full-length viral messenger RNA (mRNA), an event that occurs once every fifteen translations of the mRNA of genomic length [29]. FIV Pol, like that of primate lentiviruses, comprises the following viral enzymes: protease (PR), reverse transcriptase (RT), and integrase (IN) [28] (Figure 1). However, FIV *pol* encodes an additional protein, a deoxyuridine pyrophosphatase (DU) [30] (Figure 1).

The FIV PR is an aspartyl proteinase, which, as a functional homodimer, processes the Gag and Gag-Pol precursors into the structural proteins and enzymes of the mature virion [31]. Of note, comparison of FIV and HIV-1 PRs has shown that both enzymes have related but distinct substrate specificities [31].

After viral entry and uncoating, the FIV genomic RNA is converted by the RT into a double-stranded DNA molecule. Although the FIV RT is a polypeptide of 67 kDa, the functional enzyme is a heterodimer composed of 67-kDa and 54-kDa subunits, the latter being the PR-mediated cleavage product of the 67 kDa polypeptide [32]. The FIV RT exhibits both DNA polymerase and RNaseH activities that are essential to generating the viral cDNA and removing the viral RNA template, respectively [32]. Although the FIV RT shares with its HIV-1 counterpart a heterodimeric organization and the requirement for Mg^2+^ as a cofactor [32], it is resistant to all known non-nucleoside inhibitors (NNRTI), a biological property that has been recently explained by comparing the crystal structure of the FIV RT with that of HIV-1 [33]. Indeed, while the NNRTI binding pocket is conserved, structural differences at its entryway render the FIV RT pocket unfavorable for effective NNRTI binding [33].

The viral double-stranded DNA synthesized by the FIV RT is inserted into the chromosomes of the infected cells, thereby generating the proviral DNA. The integration process involves three steps [34]. First, the IN eliminates two nucleotides from the 3′ end of the viral DNA leaving single-stranded 5′ ends. Second, the enzyme utilizes the viral DNA 3′-hydroxyls as nucleophiles to cut the host DNA, simultaneously joining both viral DNA 3′ ends to the strands of the host DNA. Finally, the short 5′ overhangs flanking the proviral DNA are repaired by cellular enzymes. It has been shown that the FIV IN is active as a multimer, and that different functions are provided by discrete domains on different subunits [35]. A mutagenesis study of the FIV IN has revealed that this enzyme bears an N-terminal domain involved in IN multimerization and binding to the viral DNA, a central region that contains the enzyme active site, and a C-terminal domain carrying the determinants essential for IN interaction with the host DNA [35]. The viral cDNA is translocated to the nucleus as a nucleoprotein complex known as the pre-integration complex (PIC). It has been demonstrated that the cellular factor Lens Epithelium-derived Growth Factor (LEDGF/p75) is a component of the functional FIV PIC and is the main lentiviral integrase-to-chromatin tethering factor [36].

The FIV DU reduces the intracellular pool of dUTP, thereby preventing the misincorporation of dUTP instead of dTTP during the reverse transcription process, which in turn minimizes the accumulation of mutations in the viral genomic DNA [37]. DU activity is essential for FIV replication in macrophages, because these cells exhibit low levels of endogenous DU [37].

### 3.3. Rev

Transcription of the FIV proviral DNA by the cellular RNA polymerase II produces (i) multi-spliced mRNAs, such as those encoding Rev and Orf-A; (ii) single-spliced mRNAs, which code for Env and Vif; and (iii) full-length RNA species that are either used for the synthesis of the Gag and Gag-Pol precursors or packaged into virions as genomic RNA molecules [38]. After being synthesized on cytoplasmic ribosomes, the FIV Rev protein enters the nucleus and binds to the viral RNA secondary structure Rev-responsive element and to the nuclear export protein Chromosome Region Maintenance 1 (CRM1) [39]. This event allows the export to the cytoplasm of monospliced and unspliced viral RNAs.

### 3.4. Vif

Mammalian cells express apolipoprotein B mRNA-editing enzyme catalytic polypeptide-like 3 (APOBEC3) proteins, which act as restriction factors against lentivirus replication [40]. These proteins are packaged into nascent lentiviral virions and, during the next infection cycle, they deaminate cytosines to uracils during the synthesis of the cDNA strands, resulting in G-to-A hypermutation of the newly synthesized viral DNA, which is detrimental to the expression of functional viral proteins [40]. To overcome APOBEC3 antiviral action, the lentiviral Vif protein degrades host APOBEC3 proteins via the ubiquitin/proteasome-dependent pathway. Interestingly, it has been recently reported that the FIV PR cleaves feline APOBEC3 in released virions [41]. Therefore, FIV exhibits two anti-APOBEC factors, Vif and PR [41].

### 3.5. Orf-A

FIV Orf-A is a small polypeptide of 77 amino acids that is necessary for the production of infectious virions [42]. Orf-A localizes to the nucleus and promotes cell-cycle arrest of the infected cells at the G2 phase, a biological property that is also exhibited by the Vpr protein of primate lentiviruses [43]. In addition, Orf-A reduces the cell surface levels of the FIV primary receptor CD134, thereby facilitating the release of the nascent virions, which is reminiscent of the detrimental activity of the HIV-1 Nef and Vpu proteins on the expression and cell surface localization of CD4 [44].

### 3.6. Env

FIV, like the rest of the lentiviruses, encodes a single envelope glycoprotein (Env) that mediates entry of the virus into its target cells. The mature Env protein is a heterodimer composed of the surface (SU, gp95) and the transmembrane subunits (TM, gp35), which are non-covalently associated on the viral surface [45,46]. FIV cell entry requires binding of the SU glycoprotein to the cell surface receptor CD134 [47,48], which causes conformational changes in the Env protein that promote the subsequent interaction of the SU with the chemokine receptor CXCR4 [49,50]. These events are then followed by the fusion of the viral and cellular membranes, which is mediated by the TM glycoprotein [51,52,53]. It has been clearly demonstrated that one of the six variable regions of the FIV SU, the V3 domain, plays an essential role in the association of the SU glycoprotein with the viral coreceptor CXCR4 [54,55,56]. Interestingly, it has been shown that a chimeric FIV Env glycoprotein in which its SU V3 domain is replaced by the equivalent region of a CXCR4-tropic HIV-1 not only binds to CXCR4 as efficiently as wild-type FIV Env but also promotes CXCR4-dependent membrane fusion [56].

## 4. Role of the FIV Gag Domains in Virion Assembly and Budding

### 4.1. MA

As mentioned above, FIV assembles at the plasma membrane of the infected cells upon multimerization of the Gag polyprotein [21]. The FIV MA is myristoylated at the glycine in position 2 after removal of the N-terminal methionine. Insertion of the myristic acid into the lipid bilayer of the plasma membrane mediates Gag accumulation at the cell surface. Indeed, the amino acid substitution Gly2Ala in the FIV MA abrogates virion production [21]. Interestingly, analysis by electron microscopy of mammalian cells expressing this myristic-minus FIV Gag polyprotein revealed the presence of intracytoplasmic Gag shells [21]. However, FIV Gag myristoylation is necessary but not sufficient for Gag association with the plasma membrane. Another determinant in the FIV MA, composed of the three lysines at positions 26, 28, and 29, is essential for Gag localization at the plasma membrane and particle assembly [21]. In this regard, it has been shown that the HIV-1 Gag polyprotein is targeted to the cell surface by the interaction of the MA N-terminal basic patch with phosphatidylinositol-4,5-bisphosphate [PI(4,5)P2], an acidic phospholipid that is highly enriched in the inner leaflet of the plasma membrane [57]. During transport of Gag to the cell surface, the myristic acid remains sequestered within the MA domain protein structure and upon binding of the MA to PI(4,5)P2; the myristic acid is exposed, inserts into the lipid bilayer, and stabilizes the association of Gag with the plasma membrane [57]. Results of in vitro studies suggest that binding of cellular RNAs to the MA domain prevents Gag association with intracellular membranes during HIV-1 Gag trafficking to the plasma membrane [58,59]. However, when Gag molecules reach the cell surface, the PI(4,5)P2 displaces the MA-bound RNA, thereby allowing myristate exposure and Gag interaction with the plasma membrane [57]. Interestingly, depletion of PI(4,5)P2 from the plasma membrane of FIV-infected cells inhibits virion production [60]. Therefore, as observed for HIV-1, FIV also utilizes the PI(4,5)P2 for Gag targeting to the plasma membrane [60].

**Figure 3:**
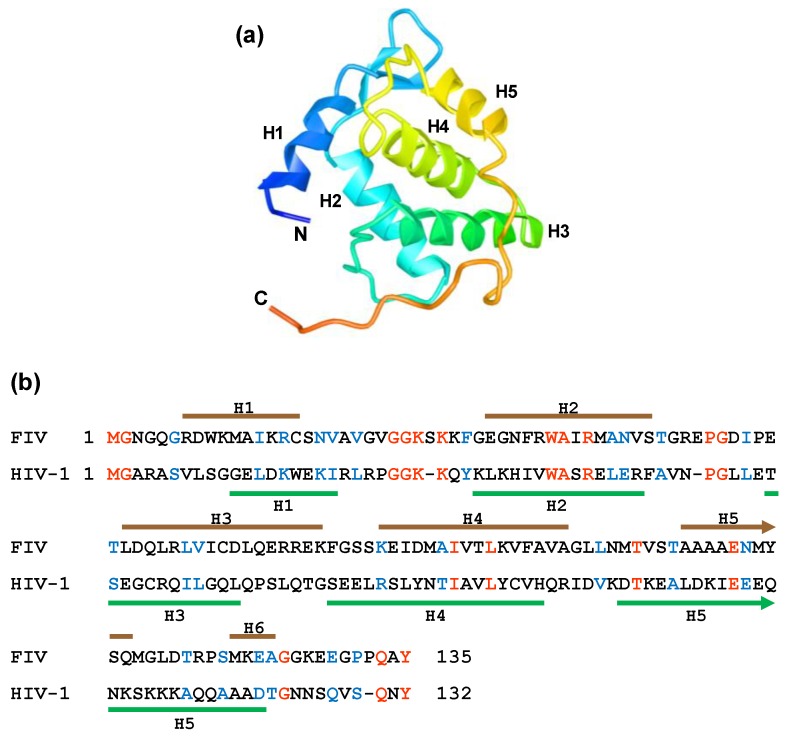
Crystal structure of the FIV MA protein (PDB 4IC9). (**a**) Ribbon diagram of the FIV MA protein in which the α-helices H1–H5 are shown together with the amino (N) and carboxyl (C) termini; (**b**) alignment of the FIV and HIV-1 MA proteins. The α-helices of the FIV MA [61] and HIV-1 MA [62] are depicted with brown and green lines, respectively. The identical amino acids at the same positions in both sequences are indicated in red, whereas the conserved residues appear in blue. The numbers indicate the length of each polypeptide.

The crystal structure of the FIV MA shows that the molecule exhibits a helical organization, typical of retroviral matrix proteins. [61] (Figure 3). However, in contrast to those of HIV-1 [62] and SIV [63], the FIV MA is not found as a trimer in the crystal [61]. In solution, the unmyristoylated FIV MA forms dimers at high protein concentration and in mildly acidic conditions [61]. Molecular docking studies have demonstrated that a myristoyl group can be placed in a hydrophobic cavity close to the side chains of residues Trp9, Phe35, Ile39, Ile53, and Phe90 [61], which is in agreement with the location of the myristic acid moiety in the NMR structure of the myristoylated FIV MA [60].

The structural and functional relationship between the MA proteins of FIV and the primate lentivirus SIV has been studied by generating chimeric proviruses, in which the MA-coding regions were exchanged. An SIV provirus containing the FIV MA domain does not produce viral particles due to the inability of the chimeric Gag polyprotein to associate with the plasma membrane [64]. This defect is reversed by increasing the basic character of the N-terminal region of the FIV MA in the chimera [64]. By contrast, replacement of the FIV MA-coding region by its SIV counterpart results in a chimeric virus that not only assembles into virions as efficiently as wild-type FIV but also replicates in a feline lymphoid cell line in a wild-type manner [64].

It has been established for both HIV-1 and SIV that the incorporation of the Env glycoprotein into virions is mediated by an interaction between the MA and the Env cytoplasmic domain [65,66,67,68]. Although mutations within the FIV Env cytoplasmic domain have been shown to block Env packaging into virions [51,53], it remains to be determined whether the FIV MA plays any role in this process.

### 4.2. CA

The CA, which is the central domain of the Gag polyprotein, plays distinct roles during lentiviral morphogenesis. As part of the Gag precursor, it participates in the protein-protein interactions that drive Gag multimerization into immature particles [27,69,70], whereas as an independent protein of the mature virion, it self-assembles into the core structure that protects the viral components required for the next steps of virus infection and spreading [71,72,73].

The CA domains of retroviral Gag polyproteins exhibit low sequence similarity, except for a 20-amino-acid motif known as the major homology region (MHR), which is highly conserved among orthoretroviruses [74]. However, comparison of the solution structures of different retroviral CA proteins shows a common organization in two highly α-helical regions that fold independently of each other: an N-terminal domain (CA-NTD) that is linked via a flexible region to a C-terminal domain (CA-CTD) [75]. Cryo-electron tomography of in vitro-assembled Gag particles from HIV-1, Mason-Pfizer monkey virus, and Rous sarcoma virus revealed that Gag assembly results in the formation of a hexagonal lattice, in which the CA-NTD organizes into hexameric rings connected by CA-CTD homodimers [76].

**Figure 4:**
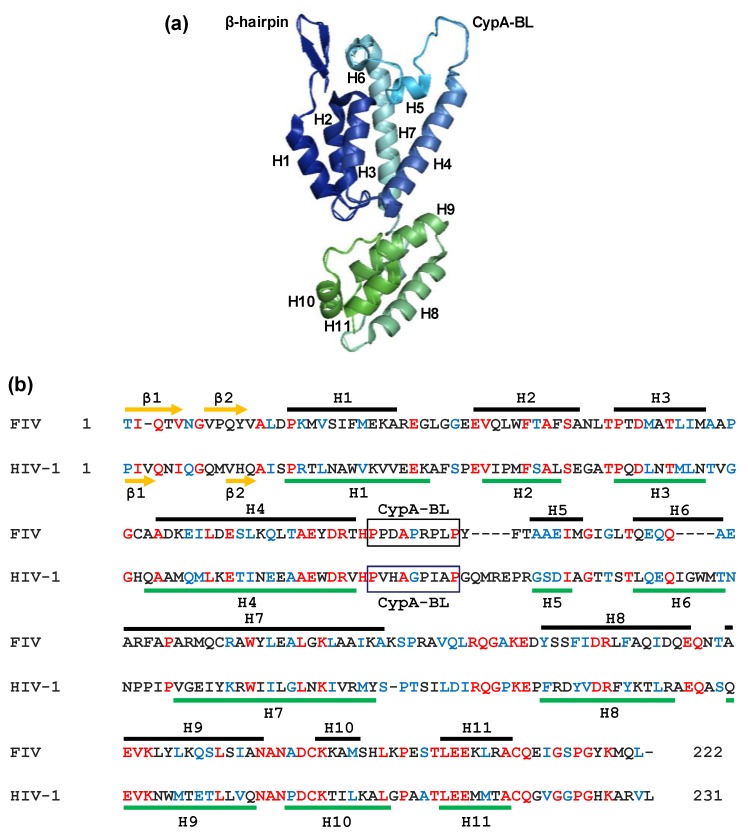
Crystal structure of the FIV CA (PDB 5NA2). (**a**) Ribbon representation of the FIV CA protein in which the α-helices H1–H7 (CA-NTD) and H8–H11 (CA-CTD) are shown together with the N-terminal β-hairpin and the CypA-binding loop (CypA-BL); (**b**) alignment of the FIV and HIV-1 CA proteins. The α-helices of the FIV CA [77] and HIV-1 CA [78] are shown as brown and green lines, respectively. The identical amino acids at the same positions in both sequences are indicated in red, whereas the conserved residues appear in blue. β1 to β2 indicate the amino acids involved in the N-terminal β-hairpin. Boxes correspond to the proposed CypA-BL. The proline at position 1 in the FIV CA sequence was replaced by a threonine residue due to technical reasons during the crystallographic procedure [77].

The recent determination of the crystal structure of the FIV CA has revealed that the molecule displays the standard α-helical CA topology with two domains separated by a linker [77] (Figure 4). However, the crystallized FIV CA shows some features that differ from known structures of lentiviral CA proteins [78,79], such as the formation of a β-hairpin motif at its amino terminal end in the absence of a proline residue at position 1 and the presence of a *cis* Arg89-Pro90 bond [77]. It has been reported that the FIV CA binds the peptidyl-prolyl *cis*-*trans* isomerase cyclophilin A (CypA) [80]. According to the crystal structure of the FIV CA, there is a CypA binding site in loop 5 [77]. Of note, among the five prolines in the FIV CA domain that CypA associates with, Pro90 is in a *cis*-conformation, which makes it a critical target for CypA due to the strong preference of this enzyme for substrates containing *cis* Pro [77]. Moreover, it has been shown that treatment of FIV-infected cells with the immunosuppressive drug cyclosporin A, which inhibits CA-CypA binding, blocks virus replication [81].

Mammalian cells express factors that inhibit retrovirus replication. In this regard, HIV-1 efficiently enters the cells of Old World monkeys but encounters a block before reverse transcription imposed by the tripartite motif protein isoform 5 alpha (TRIM5alpha), which acts directly on the incoming HIV-1 CA [82]. By contrast, HIV-1 infection is weakly restricted by human TRIM5alpha [82]. Interestingly, it is CypA that, by binding to the HIV-1 CA, isomerizes a peptide bond of this protein, making it susceptible to the restriction imposed by TRIM5alpha from Old World monkeys [83]. Of note, it has been discovered that owl monkeys exhibit a restriction factor denominated TRIMCyp that evolved from a TRIM5 protein whose C-terminal domain was replaced by CypA [84]. It has been shown that both rhesus monkey TRIM5alpha and owl monkey TRIMCyp inhibit FIV replication [85]. Potent restriction of FIV by TRIMCyp occurred in the complete absence of its RING and B-box 2 domains, whereas FIV restriction by TRIM5alpha required these regions [85]. Notably, the feline genome does not encode either TRIM5alpha or TRIMCyp proteins, and HIV-1 is primarily blocked in feline cells by APOBEC3 proteins [86].

A study aimed at identifying the FIV Gag domains that promote Gag multimerization analyzed the ability of a panel of Gag subdomains to associate with full-length Gag and be recruited into extracellular virus-like particles (VLPs). Of note, among the FIV Gag mutants tested, the CA-NC Gag subdomain associated wild-type Gag with the highest efficiency and was rescued into particles in a proportion close to 50% of the total Gag-related protein mass of VLPs [87]. Moreover, characterization of the assembly phenotype of a series of FIV Gag mutants carrying internal deletions within the CA region showed that three of the deletions that abolish Gag assembly [87] remove sequences homologous to those in HIV-1 CA helices 4, 8, and 11, which have been demonstrated to be critical for immature particle assembly [69].

Insight into the functional relationship between the SIV and FIV CA domains has been provided by two studies that characterized chimeric SIV and FIV Gag polyproteins, in which the CA domains were partially or totally exchanged [88,89]. A chimeric SIV carrying the FIV CA-NTD was found to be assembly-defective [88]. By contrast, SIVs containing the FIV CA-CTD or the entire FIV CA, p1 and the first nine residues of the FIV NC produce wild-type levels of virions that incorporate the SIV Env glycoprotein and package the viral genome RNA and RT with an efficiency similar to that of wild-type SIV [88]. However, despite being assembly-competent, the latter chimeric SIVs are non-infectious due to a post-entry impairment [88]. Furthermore, it was shown that the FIV CA-CTD dimerizes in vitro and forms high-molecular-weight oligomers, which, together with the finding that the FIV CA-CTD is sufficient to confer assembly competence to the SIV Gag polyprotein, provides evidence that the CA-CTD exhibits more functional plasticity than the CA-NTD [88]. Strikingly, chimeric FIV Gag proteins carrying different SIV CA regions are assembly-defective; however, all of them are able to interact with wild-type SIV Gag and be recruited into extracellular VLPs, regardless of the SIV CA sequences present in the chimeric FIV Gag [89].

The fact that swapping of CA regions between the SIV and FIV Gag polyproteins is reciprocally non-equivalent underscores the relevance of the other Gag domains, apart from CA for the organization and/or preservation of the Gag lattice during particle assembly. Moreover, the results from the studies described above [88,89] suggest that although lentiviral CA proteins share a similar organization, i.e., an NTD linked by a flexible region to a CTD together with a conserved MHR, their functional exchange between different lentiviruses is strictly dependent on the context of the recipient Gag precursor.

The observation that the SIV MA is biologically active in the context of FIV Gag [64], whereas SIV CA-derived sequences have a deleterious effect on FIV Gag assembly [89], is likely related to the different functions that the MA and CA domains perform during the virus life cycle.

As an additional aspect of virion formation, it should be mentioned that FIV Gag, like its HIV-1 counterpart, progresses through a pathway of assembly intermediates derived from host RNA granules, establishing interactions with the host factors ATP-binding cassette protein E1 (ABCE1), DEAD-box helicase 6 (DDX6), and mRNA Decapping protein 2 (DCP2) [90]. Interestingly, FIV CA mutants that are defective in particle production appear to be arrested at these assembly intermediates [90].

### 4.3. NC

**Figure 5:**
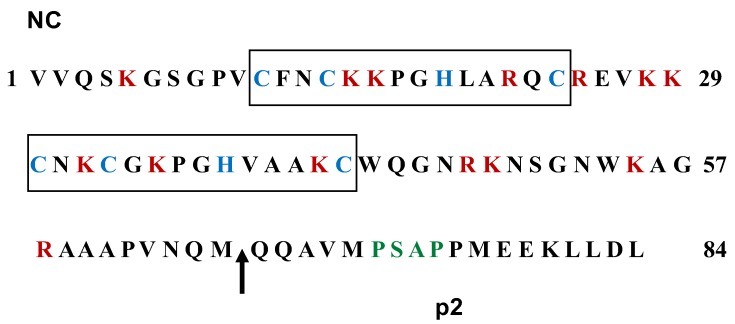
Amino acid sequences of the NC and p2 domains of FIV Gag. The proximal and distal zinc finger motifs are boxed with the characteristic cysteine and histidine residues highlighted in blue. The basic residues lysine and arginine appear in red throughout the NC sequence. The arrow indicates the PR processing site that generates the C-terminal p2 peptide. The budding domain in the p2 sequence is denoted in green. The numbers refer to the amino acid positions in the NC-p2 region.

The selective encapsidation of the full-length viral genomic RNA from a pool of cellular and viral RNAs is an essential stage in the life cycle of all retroviruses. This requires the recognition by the NC domain of the Gag polyprotein of an RNA sequence, termed encapsidation signal (E) or packaging signal (ψ), located at the 5′ end of the genome and often extending into the *gag* gene [91]. All lentiviral NC proteins exhibit a high content of basic residues and contain two copies of a zinc-binding motif with the sequence Cys-X2-Cys-X4-His-X4-Cys that is similar to those found in many DNA-binding proteins [91] (Figure 5). Analysis of the RNA binding activity and assembly phenotype of FIV NC mutant viruses has shown that substitution of serine for the first cysteine residue in the NC proximal zinc finger (Cys11Ser) is sufficient to impair both virion assembly and genomic RNA binding [92] (Figure 5). A similar defective phenotype with respect to particle formation and NC-RNA interaction is observed when the basic residues Lys28 and Lys29 in the region connecting both zinc fingers are replaced by alanine [92] (Figure 5). By contrast, mutation of the first cysteine residue in the distal zinc finger (Cys30Ser) does not affect virion production or RNA binding to the mutant NC protein [92] (Figure 5). Furthermore, the Cys30Ser NC mutant virus replicates in the feline T-cell line MYA-1 as efficiently as wild-type FIV [92]. Collectively, these data indicate that the proximal zinc finger of the FIV NC is more important for genomic RNA binding and virion assembly than the distal motif [84]. Interestingly, in HIV-1, the NC proximal Cys-His motif is also more sensitive to alteration with respect to genomic RNA packaging than the distal zinc finger [93,94].

Of note, the observation that the defect in viral RNA binding exhibited by the Cys11Ser and Lys28Ala/Lys29Ala FIV NC mutants is accompanied by a block in particle production [92] supports the concept that the NC-RNA association plays a central role in lentiviral assembly [95,96]. Further evidence for this notion is provided by the fact that the in vitro assembly of the lentiviral Gag polyproteins of HIV-1, SIV, and FIV is strictly dependent on the presence of RNA [22,25,26,27].

In addition to its role in genomic RNA packaging, chaperone properties have been attributed to the FIV NC protein such as mediating RNA dimerization, annealing of the tRNA^Lys^ to the genomic primer binding site, and minus strand DNA transfer [97].

During retrovirus assembly, two copies of the viral genomic RNA in the form of a non-covalently linked dimer are encapsidated into virions [91]. It has been shown that the FIV genome exhibits a bipartite packaging signal comprising the first 150 nucleotides of the 5′ untranslated region and the first 100 nucleotides of *gag* [98,99]. A secondary structural analysis of the FIV encapsidation region indicated that it contains four conserved stem-loops, a long-range interaction (LRI) between complementary heptanucleotides in R-U5 and *gag*, and a small palindromic stem-loop (SL5) within the *gag* open reading frame that may act as a dimerization initiation site [100]. Further study of the packaging signal led the authors to propose that the 5′ and 3′ regions previously shown to be only involved in the LRI indeed appear to alternate between two different conformations, one that favors genome-sized RNA translation and other that exposes the putative dimerization initiation site SL5, thereby promoting genome RNA packaging into virions [101].

It has been reported that the FIV CA-NC-p2 domain mediates Gag nuclear shuttling, which led the authors to suggest that FIV genomic RNA encapsidation may initiate in the nucleus [102]. However, further studies are necessary to define the exact role that Gag nuclear cycling plays in FIV replication.

### 4.4. p2

Ion spray mass spectrometry has revealed that the FIV Gag polyprotein contains a C-terminal peptide of 18 amino acids denominated p2 [28] (Figure 5). Interestingly, this peptide exhibits a PSAP motif [92], which is also found as P(S/T)AP in the C-terminal p6 domain of the HIV-1 Gag protein. It has been shown that the P(S/T)AP motif is a Gag late domain that recruits the cellular protein Tsg101 to exploit the cellular ESCRT machinery (Endosomal Sorting Complexes Required for Transport) for virus budding [103,104,105]. Interestingly, amino acid substitutions disrupting the PSAP motif in FIV Gag p2 abrogate virion production, which led to the proposal that FIV p2 is functionally equivalent to the p6 domain of HIV-1 Gag with respect to mediating virus egress [92]. Further study of FIV Gag budding has revealed that mutagenesis of the FIV Gag PSAP motif, knockdown of Tsg101 synthesis, and expression of a Tsg101 peptide that binds to the P(T/S)AP motif, each inhibits FIV release, which indicates that this process is dependent on the Tsg101-PSAP motif association [106]. It has been demonstrated that Gag PPxY motifs in Rous sarcoma virus, human T cell leukemia virus, and Mason Pfizer monkey virus bind to Nedd4, which promotes Gag ubiquitination and facilitates ESCRT recruitment [105]. In this regard, evidence has been presented that although FIV Gag lacks a PPxY motif, Nedd4-2s is capable of ubiquitinating FIV Gag, and that this process can reverse the defect in virus budding imposed by mutations in the FIV late PSAP motif [107]. However, the role of ubiquitin-ligases in FIV budding needs further investigation.

Type I interferon induces the expression at the plasma membrane of the BST2/tetherin factor that inhibits cell surface release of several enveloped viruses including retroviruses [108]. Primate lentiviruses utilize three different viral proteins to antagonize the budding restriction imposed by tetherin: Vpu (HIV-1 and Vpu-expressing SIV strains mus, gsn, and mon) [109,110], Nef (most SIV isolates) [111], and Env (HIV-2 and SIVtan) [112,113]. A feline tetherin has been identified and shown to prevent the release of FIV virions in transient assays, which led to the suggestion that FIV may lack a tetherin antagonist [114]. However, viral replication was found to occur in the presence of tetherin due to FIV cell-to-cell transmission mediated by syncytium formation [114]. In contrast, other studies have shown that feline tetherin fails to restrict wild-type FIV budding, and that the egress of Env-defective FIV mutants is blocked [115,116]. The budding defect exhibited by these *env*-minus mutants can be reversed by transient expression of the FIV Env protein, which indicates that feline cells express a functional tetherin, and that the FIV Env glycoprotein is the viral factor counteracting tetherin restriction [115,116].

## 5. Conclusions

In this review, we summarize the available information about the different steps in FIV assembly and budding, as well as the specific contributions of each of the Gag domains to these processes. We highlight the parallels and distinctions between FIV morphogenesis and that of primate lentiviruses to illustrate how the molecular mechanisms underlying the generation of infectious virions have evolved in lentiviruses. In-depth knowledge of FIV biology is essential for the development of broad antiviral strategies aimed at blocking or interfering with lentiviral replication.

## References

[1] Pedersen N.C., Ho E.W., Brown M.L., Yamamoto J.K. (1987). Isolation of a T-lymphotropic virus from domestic cats with an immunodeficiency-like syndrome. Science.

[2] White J., Stickney A., Norris J.M. (2011). Feline immunodeficiency virus: Disease association versus causation in domestic and nondomestic felids. Vet. Clin..

[3] O’Brien S.J., Troyer J.L., Brown M.A., Johnson W.E., Antunes A., Roelke M.E., Pecon-Slattery J. (2012). Emerging viruses in the Felidae: Shifting paradigms. Viruses.

[4] Matteucci D., Baldinotti F., Mazzetti P., Pistello M., Bandecchi P., Ghilarducci R., Poli A., Tozzini F., Bendinelli M. (1993). Detection of feline immunodeficiency virus in saliva and plasma by cultivation and polymerase chain reaction. J. Clin. Microbiol..

[5] Elder J.H., Lin Y.C., Fink E., Grant C.K. (2010). Feline immunodeficiency virus (FIV) as a model for study of lentivirus infections: Parallels with HIV. Curr. HIV Res..

[6] Allison R.W., Hoover E.A. (2003). Covert vertical transmission of feline immunodeficiency virus. AIDS Res. Hum. Retrovir..

[7] Brown W.C., Bissey L., Logan K.S., Pedersen N.C., Elder J.H., Collisson E.W. (1991). Feline immunodeficiency virus infects both CD4+ and CD8+ T lymphocytes. J. Virol..

[8] Brunner D., Pedersen N.C. (1989). Infection of peritoneal macrophages in vitro and in vivo with feline immunodeficiency virus. J. Virol..

[9] Dean G.A., Himathongkham S., Sparger E.E. (1999). Differential cell tropism of feline immunodeficiency virus molecular clones in vivo. J. Virol..

[10] English R.V., Johnson C.M., Gebhard D.H., Tompkins M.B. (1993). In vivo lymphocyte tropism of feline immunodeficiency virus. J. Virol..

[11] Kenyon J.C., Lever A.M. (2011). The molecular biology of feline immunodeficiency virus (FIV). Viruses.

[12] Burkhard M., Dean G.A. (2003). Transmission and immunopathogenesis of FIV in cats as a model for HIV. Curr. HIV Res..

[13] Sparger E.E. (2006). FIV as a model for HIV: An overview. In Vivo Models of HIV Disease and Control.

[14] Meeker R.B., Hudson L. (2017). Feline immunodeficiency virus neuropathogenesis: A model for HIV-induced CNS inflammation and neurodegeneration. Vet. Sci..

[15] Arai M., Earl D.D., Yamamoto J.K. (2002). Is AZT/3TC therapy effective against FIV infection or immunopathogenesis?. Vet. Immunol. Immunopathol..

[16] Hayes K.A., Lafrado L.J., Erickson J.G., Marr J.M., Mathes L.E. (1993). Prophylactic ZDV therapy prevents early viremia and lymphocyte decline but not primary infection in feline immunodeficiency virus-inoculated cats. J. Acquir. Immune Defic. Syndr..

[17] Egberink H., Borst M., Niphuis H., Balzarini J., Neu H., Schellekens H., de Clercq E., Horzinek M., Koolen M. (1990). Suppression of feline immunodeficiency virus infection in vivo by 9-(2-phosphonomethoxyethyl)adenine. Proc. Natl. Acad. Sci. USA.

[18] Hartmann K., Donath A., Beer B., Egberink H.F., Horzinek M.C., Lutz H., Hoffmann-Fezer G., Thum I., Thefeld S. (1992). Use of two virustatica (AZT, PMEA) in the treatment of FIV and of FeLV seropositive cats with clinical symptoms. Vet. Immunol. Immunopathol..

[19] Savarino A., Pistello M., D’Ostilio D., Zabogli E., Taglia F., Mancini F., Ferro S., Matteucci D., de Luca L., Barreca M.L. (2007). Human immunodeficiency virus integrase inhibitors efficiently suppress feline immunodeficiency virus replication in vitro and provide a rationale to redesign antiretroviral treatment for feline AIDS. Retrovirology.

[20] Olmsted R.A., Hirsch V.M., Purcell R.H., Johnson P.R. (1989). Nucleotide sequence analysis of feline immunodeficiency virus: Genome organization and relationship to other lentiviruses. Proc. Natl. Acad. Sci. USA.

[21] Manrique M.L., Celma C.C., González S.A., Affranchino J.L. (2001). Mutational analysis of the feline immunodeficiency virus matrix protein. Virus Res..

[22] Affranchino J.L., González S.A. (2010). In vitro assembly of feline immunodeficiency virus Gag polyprotein. Virus Res..

[23] Göttlinger H.G., Sodroski J.G., Haseltine W.A. (1989). Role of capsid precursor processing and myristoylation in morphogenesis and infectivity of human immunodeficiency virus type 1. Proc. Natl. Acad. Sci. USA.

[24] González S.A., Affranchino J.L., Gelderblom H.R., Burny A. (1993). Assembly of the matrix protein of simian immunodeficiency virus into virus-like particles. Virology.

[25] Campbell S., Rein A. (1999). In vitro assembly properties of human immunodeficiency virus type 1 Gag protein lacking the p6 domain. J. Virol..

[26] Huseby D., Barklis R.L., Alfadhli A., Barklis E. (2005). Assembly of human immunodeficiency virus precursor Gag proteins. J. Biol. Chem..

[27] Rauddi M.L., Mac Donald C.L., Affranchino J.L., González S.A. (2011). Mapping of the self-interaction domains in the simian immunodeficiency virus Gag polyprotein. AIDS Res. Hum. Retrovir..

[28] Elder J.H., Schnölzer M., Hasselkus-Light C.S., Henson M., Lerner D.A., Phillips T.R., Wagaman P.C., Kent S.B. (1993). Identification of proteolytic processing sites within the Gag and Pol polyproteins of feline immunodeficiency virus. J. Virol..

[29] Morikawa S., Bishop D.H. (1992). Identification and analysis of the gag-pol ribosomal frameshift site of feline immunodeficiency virus. Virology.

[30] Elder J.H., Lerner D.L., Hasselkus-Light C.S., Fontenot D.J., Hunter E., Luciw P.A., Montelaro R.C., Phillips T.R. (1992). Distinct subsets of retroviruses encode dUTPase. J. Virol..

[31] Schnölzer M., Rackwitz H.R., Gustchina A., Laco G.S., Wlodawer A., Elder J.H., Kent S.B. (1996). Comparative properties of feline immunodeficiency virus (FIV) and human immunodeficiency virus type 1 (HIV-1) proteinases prepared by total chemical synthesis. Virology.

[32] North T.W., Cronn R.C., Remington K.M., Tandberg R.T., Judd R.C. (1990). Characterization of reverse transcriptase from feline immunodeficiency virus. J. Biol. Chem..

[33] Galilee M., Alian A. (2018). The structure of FIV reverse transcriptase and its implications for non-nucleoside inhibitor resistance. PLoS Pathog..

[34] Lesbats P., Engelman A.N., Cherepanov P. (2016). Retroviral DNA Integration. Chem. Rev..

[35] Shibagaki Y., Holmes M.L., Appa R.S., Chow S.A. (1997). Characterization of feline immunodeficiency virus integrase and analysis of functional domains. Virology.

[36] Llano M., Vanegas M., Fregoso O., Saenz D., Chung S., Peretz M., Poeschla E.M. (2004). LEDGF/p75 determines cellular trafficking of diverse lentiviral but not murine oncoretroviral integrase proteins and is a component of functional lentiviral preintegration complexes. J. Virol..

[37] Lerner D.L., Wagaman P.C., Phillips T.R., Prospero-Garcia O., Henriksen S.J., Fox H.S., Bloom F.E., Elder J.H. (1995). Increased mutation frequency of feline immunodeficiency virus lacking functional deoxyuridine-triphosphatase. Proc. Natl. Acad. Sci. USA.

[38] Tomonaga K., Shin Y.S., Fukasawa M., Miyazawa T., Adachi A., Mikami T. (1993). Feline immunodeficiency virus gene expression: Analysis of the RNA splicing pattern and the monocistronic rev mRNA. J. Gen. Virol..

[39] Neville M., Stutz F., Lee L., Davis L.I., Rosbash M. (1997). The importin-beta family member Crm1p bridges the interaction between Rev and the nuclear pore complex during nuclear export. Curr. Biol..

[40] Desimmie B.A., Delviks-Frankenberrry K.A., Burdick R.C., Qi D., Izumi T., Pathak V.K. (2014). Multiple APOBEC3 restriction factors for HIV-1 and one Vif to rule them all. J. Mol. Biol..

[41] Yoshikawa R., Takeuchi J.S., Yamada E., Nakano Y., Misawa N., Kimura Y., Ren F., Miyazawa T., Koyanagi Y., Sato K. (2017). Feline immunodeficiency virus evolutionarily acquires two proteins, Vif and Protease, capable of antagonizing feline APOBEC3. J. Virol..

[42] Gemeniano M.C., Sawai E.T., Leutenegger C.M., Sparger E.E. (2003). Feline immunodeficiency virus ORF-A is required for virus particle formation and virus infectivity. J. Virol..

[43] Gemeniano M.C., Sawai E.T., Sparger E.E. (2004). Feline immunodeficiency virus ORF-A localizes to the nucleus and induces cell cycle arrest. Virology.

[44] Hong Y., Fink E., Hu Q.Y., Kiosses W.B., Elder J.H. (2010). ORF-A downregulates feline immunodeficiency virus primary receptor CD134 on the host cell surface and is important in viral infection. J. Virol..

[45] Verschoor E.J., Hulskotte E.G.J., Ederveen J., Koolen M.J.M., Horzinek M.C., Rottier P.J.M. (1993). Post-translational processing of the feline immunodeficiency virus envelope precursor protein. Virology.

[46] Affranchino J.L., González S.A. (2014). Understanding the process of envelope glycoprotein incorporation into virions in simian and feline immunodeficiency viruses. Viruses.

[47] Shimojima M., Miyasawa T., Ikeda Y., McMonagle E.L., Haining H., Akashi H., Takeuchi Y., Hosie M.J., Willett B.J. (2004). Use of CD134 as a primary receptor by the feline immunodeficiency virus. Science.

[48] De Parseval A., Chatterji U., Sun P., Elder J.H. (2004). Feline immunodeficiency virus targets activated CD4+ T cells by using CD134 as a binding receptor. Proc. Natl. Acad. Sci. USA.

[49] Poeschla E.M., Looney D.J. (1998). CXCR4 is required by a nonprimate lentivirus: Heterologous expression of feline immunodeficiency virus in human, rodent, and feline cells. J. Virol..

[50] Willett B.J., Picard L., Hosie M.J., Turner J.D., Adema K., Clapham P.R. (1997). Shared usage of the chemokine receptor CXCR4 by the feline and human immunodeficiency viruses. J. Virol..

[51] Celma C.C.P., Paladino M.G., González S.A., Affranchino J.L. (2007). Importance of the short cytoplasmic domain of feline immunodeficiency virus transmembrane glycoprotein for fusion activity and envelope glycoprotein incorporation into virions. Virology.

[52] Garg H., Fuller F.J., Tompkins W.A.F. (2004). Mechanism of feline immunodeficiency virus envelope glycoprotein-mediated fusion. Virology.

[53] González S.A., Paladino M.G., Affranchino J.L. (2012). Palmitoylation of the feline immunodeficiency virus envelope glycoprotein and its effect on fusion activity and envelope incorporation into virions. Virology.

[54] Hu Q.Y., Fink E., Hong Y., Wang C., Grant C.K., Elder J.H. (2010). Fine definition of the CXCR4-binding region on the V3 loop of feline immunodeficiency virus surface glycoprotein. PLoS ONE.

[55] Sundstrom M., White R.L., de Parseval A., Sastry K.J., Morris G., Grant C.K., Elder J.H. (2008). Mapping of the CXCR4 binding site within variable region 3 of the feline immunodeficiency virus surface glycoprotein. J. Virol..

[56] González S.A., Falcón J.I., Affranchino J.L. (2014). Replacement of the V3 domain in the surface subunit of the feline immnodeficiency virus envelope glycoprotein with the equivalent region of a T cell-tropic human immunodeficiency virus type 1 results in a chimeric surface protein that efficiently binds to CXCR4. AIDS Res. Hum. Retrovir..

[57] Freed E.O. (2015). HIV-1 assembly, release and maturation. Nat. Rev. Microbiol..

[58] Alfadhli A., Still A., Barklis E. (2009). Analysis of human immunodeficiency virus type 1 matrix binding to membranes and nucleic acids. J. Virol..

[59] Chukkapalli V., Oh S.J., Ono A. (2010). Opposing mechanisms involving RNA and lipids regulate HIV-1 Gag membrane binding through the highly basic region of the matrix domain. Proc. Natl Acad. Sci. USA.

[60] Brown L.A., Cox C., Baptiste J., Summers H., Button R., Bahlow K., Spurrier V., Kyser J., Luttge B.G., Kuo L. (2015). NMR structure of the myristylated feline immunodeficiency virus matrix protein. Viruses.

[61] Serrière J., Robert X., Perez M., Gouet P., Guillon C. (2013). Biophysical characterization and crystal structure of the feline immunodeficiency virus p15 matrix protein. Retrovirology.

[62] Hill C.P., Worthylake D., Bancroft D.P., Christensen A.M., Sundquist W.I. (1996). Crystal structures of the trimeric human immunodeficiency virus type 1 matrix protein: Implications for membrane association and assembly. Proc. Natl. Acad. Sci. USA.

[63] Rao Z., Belyaev A.S., Fry E., Roy P., Jones I.M., Stuart D.I. (1995). Crystal structure of SIV matrix antigen and implications for virus assembly. Nature.

[64] Manrique M.L., González S.A., Affranchino J.L. (2004). Functional relationship between the matrix proteins of feline and simian immunodeficiency viruses. Virology.

[65] Freed E.O., Martin M.A. (1996). Domains of the human immunodeficiency virus type 1 matrix and gp41 cytoplasmic tail required for envelope incorporation into virions. J. Virol..

[66] Murakami T., Freed E.O. (2000). Genetic evidence for an interaction between human immunodeficiency virus type 1 matrix and α-helix 2 of the gp41 cytoplasmic tail. J. Virol..

[67] Manrique J.M., Celma C.C., Hunter E., Affranchino J.L., González S.A. (2003). Positive and negative modulation of virus infectivity and envelope glycoprotein incorporation into virions by amino acid substitutions at the N terminus of the simian immunodeficiency virus matrix protein. J. Virol..

[68] Manrique J.M., Affranchino J.L., González S.A. (2008). In vitro binding of simian immunodeficiency virus matrix protein to the cytoplasmic domain of the envelope glycoprotein. Virology.

[69] Von Schwedler U.K., Stray K.M., Garrus J.E., Sundquist W.I. (2003). Functional surfaces of the human immunodeficiency virus type 1 capsid protein. J. Virol..

[70] Bharat T.A., Davey N.E., Ulbrich P., Riches J.D., de Marco A., Rumlova M., Sachse C., Ruml T., Briggs J.A. (2012). Structure of the immature retroviral capsid at 8Å resolution by cryo-electron microscopy. Nature.

[71] Forshey B.M., von Schwedler U., Sundquist W.I., Aiken C. (2002). Formation of a human immunodeficiency virus type 1 core of optimal stability is crucial for viral replication. J. Virol..

[72] Briggs J.A., Wilk T., Welker R., Kräusslich H.G., Fuller S.D. (2003). Structural organization of authentic, mature HIV-1 virions and cores. EMBO J..

[73] Woodward C.L., Cheng S.N., Jensen G.J. (2015). Electron cryotomography studies of maturing HIV-1 particles reveal the assembly pathway of the viral core. J. Virol..

[74] Wills J.W., Craven R.C. (1991). Form, function, and use of retroviral gag proteins. AIDS.

[75] Campos-Olivas R., Newman J.L., Summers M.F. (2000). Solution structure and dynamics of the Rous sarcoma virus capsid protein and comparison with capsid proteins of other retroviruses. J. Mol. Biol..

[76] De Marco A., Davey N.E., Ulbrich P., Phillips J.M., Lux V., Riches J.D., Fuzik T., Ruml T., Kräusslich H.G., Vogt V.M. (2010). Conserved and variable features of Gag structure and arrangement in immature retrovirus particles. J. Virol..

[77] Folio C., Sierra N., Dujardin M., Alvarez G., Guillon C. (2017). Crystal structure of the full-length feline immunodeficiency virus capsid protein shows an N-terminal β-hairpin in the absence of N-terminal proline. Viruses.

[78] Gres A.T., Kirby K.A., KewalRamani V.N., Tanner J.J., Pornillos O., Sarafianos S.G. (2015). Structural virology. X-ray crystal structures of native HIV-1 capsid protein reveal conformational variability. Science.

[79] Jin Z., Jin L., Peterson D.L., Lawson C.L. (1999). Model for lentivirus capsid core assembly based on crystal dimers of EIAV p26. J. Mol. Biol..

[80] Lin T.Y., Emerman M. (2006). Cyclophilin A interacts with diverse lentiviral capsids. Retrovirology.

[81] Mortola E., Endo Y., Ohno K., Watari T., Tsujimoto H., Hasegawa A. (1998). The use of two immunosuppressive drugs, *Cyclosporin* A and tacrolimus, to inhibit virus replication and apoptosis in cells infected with feline immunodeficiency virus. Vet. Res. Commun..

[82] Stremlau M., Owens C.M., Perron M.J., Kiessling M., Autissier P., Sodroski J. (2004). The cytoplasmic body component TRIM5α restricts HIV-1 infection in Old World monkeys. Nature.

[83] Towers G.J. (2007). The control of viral infection by tripartite motif proteins and Cyclophilin A. Retrovirology.

[84] Sayah D.M., Sokolskaja E., Berthoux L., Luban J. (2004). Cyclophilin A retrotransposition into TRIM5 explains owl monkey resistance to HIV-1. Nature.

[85] Diaz-Griffero F., Kar A., Lee M., Stremlau M., Poeschla E., Sodroski J. (2007). Comparative requirements for the restriction of retrovirus infection by TRIM5α and TRIMCyp. Virology.

[86] Poeschla E.M. (2011). Primate and feline lentiviruses in current intrinsic immunity research: The cat is back. Vet. Immunol. Immunopathol..

[87] Abdusetir Cerfoglio J.C., González S.A., Affranchino J.L. (2014). Structural elements in the Gag polyprotein of feline immunodeficiency virus involved in Gag self-association and assembly. J. Gen. Virol..

[88] Esteva M.J., Affranchino J.L., González S.A. (2014). Lentiviral Gag assembly analyzed through the functional characterization of chimeric simian immunodeficiency viruses expressing different domains of the feline immunodeficiency virus capsid protein. PLoS ONE.

[89] Ovejero C.A., Affranchino J.L., González S.A. (2017). Analysis of the functional compatibility of SIV capsid sequences in the context of the FIV Gag precursor. PLoS ONE.

[90] Reed J.C., Westergreen N., Barajas B.C., Ressler D.T.B., Phuong D.J., Swain J.V., Lingappa V.R., Lingappa J.R. (2018). The formation of RNA granule-derived capsid assembly intermediates appears to be conserved between HIV-1 and the non-primate lentivirus FIV. J. Virol..

[91] Jewell N.A., Mansky L.M. (2000). In the beginning: Genome recognition, RNA encapsidation and the initiation of complex retrovirus assembly. J. Gen. Virol..

[92] Manrique M.L., Rauddi M.L., González S.A., Affranchino J.L. (2004). Functional domains in the feline immunodeficiency virus nucleocapsid protein. Virology.

[93] Gorelick R.J., Chabot D.J., Rein A., Henderson L.E., Arthur L.O. (1993). The two zinc fingers in the human immunodeficiency virus type 1 nucleocapsid protein are not functionally equivalent. J. Virol..

[94] Schwartz M.D., Fiore D., Panganiban A.T. (1997). Distinct functions and requirements for the Cys-His boxes of the human immunodeficiency virus type 1 nucleocapsid protein during RNA encapsidation and replication. J. Virol..

[95] Zhang Y., Qian H., Love Z., Barklis E. (1998). Analysis of the assembly function of the human immunodeficiency virus type 1 gag protein nucleocapsid domain. J. Virol..

[96] Muriaux D., Mirro J., Harvin D., Rein A. (2001). RNA is a structural element in retrovirus particles. Proc. Natl. Acad. Sci. USA.

[97] Moscardini M., Pistello M., Bendinelli M., Ficheux D., Miller J.T., Gabus C., Le Grice S.F.J., Surewicz W.K., Darlix J.L. (2002). Functional interactions of nucleocapsid protein of feline immunodeficiency virus and cellular prion protein with viral RNA. J. Mol. Biol..

[98] Kemler I., Barraza R., Poeschla E.M. (2002). Mapping the encapsidation determinants of feline immunodeficiency virus. J. Virol..

[99] Browning M.T., Mustafa F., Schmidt R.D., Lew K.A., Rizvi T.A. (2003). Delineation of sequences important for efficient packaging of feline immunodeficiency virus RNA. J. Gen. Virol..

[100] Kenyon J.C., Ghazawi A., Cheung W.K., Phillip P.S., Rizvi T.A., Lever A.M. (2008). The secondary structure of the 5′ end of the FIV genome reveals a long-range interaction between R/U5 and gag sequences, and a large, stable stem-loop. RNA.

[101] Kenyon J.C., Tanner S.J., Legiewicz M., Phillip P.S., Rizvi T.A., Le Grice S.F.J., Lever A.M.L. (2011). SHAPE analysis of the FIV leader RNA reveals a structural switch potentially controlling viral packaging and genome dimerization. Nucleic Acids Res..

[102] Kemler I., Saenz D., Poeschla E. (2012). Feline immunodeficiency virus Gag is a nuclear shuttling protein. J. Virol..

[103] VerPlank L., Bouamr F., LaGrassa T.J., Agresta B., Kikonyogo A., Leis J., Carter C.A. (2001). Tsg101, a homologue of ubiquitin-conjugating (E2) enzymes, binds the L domain in HIV type 1 Pr55(Gag). Proc. Natl. Acad. Sci. USA.

[104] Martin-Serrano J., Zang T., Bieniasz P.D. (2001). HIV-1 and Ebola virus encode small peptide motifs that recruit Tsg101 to sites of particle assembly to facilitate egress. Nat. Med..

[105] Votteler J., Sundquist W.I. (2013). Virus budding and the ESCRT pathway. Cell Host Microbe.

[106] Luttge B.G., Shehu-Xhilaga M., Demirov D.G., Adamson C.S., Soheilian F., Nagashima K., Stephen A.G., Fisher R.J., Freed E.O. (2008). Molecular characterization of feline immunodeficiency virus budding. J. Virol..

[107] Calistri A., Del Vecchio C., Salata C., Celestino M., Celegato M., Göttlinger H., Palù G., Parolin C. (2009). Role of the feline immunodeficiency virus L-domain in the presence or absence of Gag processing: Involvement of ubiquitin and Nedd4-2s ligase in viral egress. J. Cell Physiol..

[108] Jouvenet N., Neil S.J., Zhadina M., Zang T., Kratovac Z., Lee Y., McNatt M., Hatziioannou T., Bieniasz P.D. (2009). Broad-spectrum inhibition of retroviral and filoviral particle release by tetherin. J. Virol..

[109] Neil S.J., Zang T., Bieniasz P.D. (2008). Tetherin inhibits retrovirus release and is antagonized by HIV-1 Vpu. Nature.

[110] Yang S.J., Lopez L.A., Hauser H., Exline C.M., Haworth K.G., Cannon P.M. (2010). Anti-tetherin activities in Vpu-expressing primate lentiviruses. Retrovirology.

[111] Zhang F., Wilson S.J., Landford W.C., Virgen B., Gregory D., Johnson M.C., Munch J., Kirchhoff F., Bieniasz P.D., Hatziioannou T. (2009). Nef proteins from simian immunodeficiency viruses are tetherin antagonists. Cell Host Microbe.

[112] Le Tortorec A., Neil S.J. (2009). Antagonism to and intracellular sequestration of human tetherin by the human immunodeficiency virus type 2 envelope glycoprotein. J. Virol..

[113] Gupta R.K., Mlcochova P., Pelchen-Matthews A., Petit S.J., Mattiuzzo G., Pillay D., Takeuchi Y., Marsh M., Towers G.J. (2009). Simian immunodeficiency virus envelope glycoprotein counteracts tetherin/BST-2/CD317 by intracellular sequestration. Proc. Natl. Acad. Sci. USA.

[114] Dietrich I., McMonagle E.L., Petit S.J., Vijayakrishnan S., Logan N., Chan C.N., Towers G.J., Hosie M.J., Willett B.J. (2011). Feline tetherin efficiently restricts release of feline immunodeficiency virus but not spreading of infection. J. Virol..

[115] Celestino M., Calistri A., Del Vecchio C., Salata C., Chiuppesi F., Pistello M., Borsetti A., Palù G., Parolin C. (2012). Feline tetherin is characterized by a short N-terminal region and is counteracted by the feline immunodeficiency virus envelope glycoprotein. J. Virol..

[116] Morrison J.H., Guevara R.B., Marcano A.C., Saenz D.T., Fadel H.J., Rogstad D.K., Poeschla E.M. (2014). Feline immunodeficiency virus envelope glycoproteins antagonize tetherin through a distinctive mechanism that requires virion incorporation. J. Virol..

